# Does lower gastrointestinal endoscopy during pregnancy pose a risk for mother and child? – a systematic review

**DOI:** 10.1186/s12876-015-0244-z

**Published:** 2015-02-12

**Authors:** Alison De Lima, Boris Galjart, Pieter HA Wisse, Wichor M Bramer, C Janneke van der Woude

**Affiliations:** 1 Department of Gastroenterology and Hepatology, Erasmus MC - University Medical Center Rotterdam, ‘s Gravendijkwal 230, Room Hs-306, 3015 CE Rotterdam, The Netherlands; 2 Medical Library, Erasmus MC – University Medical Center Rotterdam, Rotterdam, The Netherlands

**Keywords:** Endoscopy, Pregnancy, Colonoscopy, Sigmoidoscopy

## Abstract

**Background:**

Gastrointestinal endoscopy plays a crucial role in the diagnosis and management of gastrointestinal disorders. When endoscopy is indicated during pregnancy, concerns about the effects on pregnancy outcome often arise. The aim of this study was to assess whether lower gastrointestinal endoscopies (LGEs) across all three trimesters of pregnancy affects pregnancy outcomes.

**Methods:**

A systematic literature search was performed using Embase (including MEDLINE), Medline OvidSP, Cochrane Central Register of Controlled Trials, Web-of-Science, Google scholar and Pubmed. All original research articles from 1990 until May 2014 involving pregnant women who underwent LGE for any indication were included. Adverse pregnancy events like spontaneous abortion, preterm birth and fetal demise were assessed for a temporal and etiological relation with the LGE.

**Results:**

In total, 5514 references were screened by two independent reviewers. Eighty-two references met the inclusion criteria and were selected. Two retrospective, controlled studies, one uncontrolled study and 79 case reports were identified. In the three studies, birth outcomes did not differ between women undergoing LGE during pregnancy, compared to women that had an indication for LGE but in whom LGE was not performed because of pregnancy. In 79 case reports, 92 patients are described who underwent 100 LGE’s during pregnancy. LGEs performed in all trimesters (n = 32, 39 and 29) were both temporally and etiologically related to 1, 3 and 2 adverse events, respectively.

**Conclusion:**

Based on the available literature, this review concludes that lower gastrointestinal endoscopy during pregnancy is of low risk for mother and child in all three trimesters of pregnancy.

**Electronic supplementary material:**

The online version of this article (doi:10.1186/s12876-015-0244-z) contains supplementary material, which is available to authorized users.

## Background

Gastrointestinal endoscopy plays a crucial role in the diagnosis and management of acute and chronic gastrointestinal disorders. In general, sigmoidoscopy and colonoscopy are regarded of low risk, because of the very low rate of serious complications following lower gastrointestinal endoscopy (LGE) [1,2]. Endoscopic procedures during pregnancy are less common, and although an estimated 6000 pregnant women in the United States annually have an indication for endoscopy, the safety of endoscopy during pregnancy remains unknown [3]. LGE during pregnancy raises important safety questions, including whether medication or bowel preparation is associated with placental abruption or fetal trauma during endoscopic intubation [4] and fetal demise due to maternal hypoxia [4], hypotension or cardiac arrhythmias [5]. Despite the paucity of data, oesophagoduodenoscopy [6,7] and sigmoidoscopy [8] are considered relatively safe during pregnancy. The safety of colonoscopy during pregnancy remains more elusive and under debate. In recent ASGE guidelines LGE is regarded of low risk during pregnancy, and it is concluded that if possible this should be deferred to the second trimester [9]. Recently, we had to perform several endoscopies in other trimesters and therefore we decided to perform a systematic literature search to assess the effect of the timing of LGE during pregnancy on adverse pregnancy outcomes like spontaneous abortion, stillbirth and premature labor.

## Methods

### Search strategy

A systematic database search for citations about LGE during pregnancy was performed by the first author (ADL) and an information specialist (WMB) on May 26th 2014. This search was performed in the following databases: Embase (including MEDLINE), Medline OvidSP, Cochrane Central Register of Controlled Trials, Web-of-Science, Google scholar and Pubmed. The detailed digital search strategy is provided in the Additional file 1.

### Review and study selection process

Titles and abstracts identified through the search strategy were assessed by two independent reviewers for potential eligibility. All original research articles, including case reports, were included. References were excluded on title and abstract based on the following exclusion criteria: all references published before 1990, all references not in English, all references regarding different subjects, conference proceedings and animal studies. Disagreements were settled in consensus and, if necessary, after discussion with a third independent reviewer. The manuscripts deemed potentially eligible for inclusion were obtained for full text review. The full texts were assessed by the two independent reviewers ((1) ADL and (2) BG and PHAW), using pre-defined eligibility criteria. Articles were included when the study population consisted of at least one pregnant female and LGE was performed during pregnancy. Articles on ectopic pregnancy were excluded, as well as articles without outcome information on the mother and the child. Discussions with the third independent (CJW) reviewer were used to resolve disagreements.

### Data extraction

Data from the eligible reports was extracted using a standardized form by the primary reviewers. Differences in the extracted data were resolved through consensus or, if necessary, discussion with the third independent reviewer. For each study, the following data was extracted considering the following:

1. Procedure (type of endoscopy, gestational week of endoscopy)
2. Participants (including age, indication for endoscopy)
3. Interventions (additional surgery, medical treatment, gestational week of other interventions)
4. Outcomes (including birth outcomes, fetal adverse events, maternal adverse events, gestational week of adverse events)

### Definitions

Sigmoidoscopy was defined as endoscopic intubation no further than the splenic flexure, and colonoscopy was defined as endoscopic intubation beyond the splenic flexure.

Miscarriages or spontaneous abortion were defined as fetal loss prior to 20th gestational week. Stillbirth or fetal demise was defined as fetal loss beyond the 20th gestational week. Premature delivery was defined as delivery before gestational week 37.

A temporal relation between an adverse event and LGE was found as plausible if the adverse event occurred within 1 week of the LGE and defined as unlikely when the adverse event occurred more than 1 week after endoscopy.

An etiological relation was found plausible if a temporal relation existed and, in addition, based on sound, medical reasoning the adverse event could be linked to the LGE. Etiological relations were classified on an ordinal scale as: unlikely, possible, probable and likely. These relations were determined in consensus, based on the following definitions.

Unlikely relation: LGE or its preparation or sedation cannot explain maternal/fetal adverse event, based on sound, medical reasoning. Elective abortions and induced labor or elective caesarean sections were all classified as unlikely related to LGE.

Possible relation: LGE or its preparation or sedation could explain maternal/fetal adverse event, however in between LGE and the occurrence of the adverse event another intervention was also performed (e.g. laparotomy).

Probable relation: LGE or its preparation or sedation could explain maternal/fetal adverse event, no other interventions between LGE and adverse event were performed, however, the underlying maternal disease could still also explain the adverse event.

Likely relation: LGE or its preparation or sedation could explain maternal/fetal adverse event, no other interventions between adverse event were performed, maternal disease does not seem etiologically related to the adverse event.

## Results

**Figure 1:**
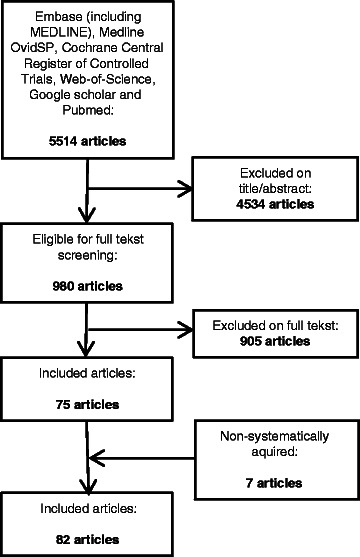
**Flowchart of study selection process.**

The search yielded a total of 5514 citations. After reviewing title and abstracts, 980 manuscripts were selected for further review. After review of the full text, 75 articles were included, including one retrospective uncontrolled study, two retrospective, controlled studies and 72 case reports or series. An additional non-systematic search yielded another 7 case reports, resulting in a total of 82 articles (See flowchart in Figure 1).

### Description of the studies

An uncontrolled, retrospective, multicenter study in 1995 [10], which was published again one year later as an expanded cohort with added controls [8], reported 46 pregnant patients undergoing 48 sigmoidoscopies and 8 pregnant patients undergoing 8 colonoscopies. There were no differences in birth outcomes between the pregnant patients undergoing endoscopy during pregnancy compared to the pregnant patients not undergoing endoscopy during pregnancy. Both groups had similar indications for endoscopy. In addition, there were no differences in birth outcomes compared to the national American rates at that time. No adverse maternal events were reported following endoscopy. Following sigmoidoscopy, 4 voluntary abortions and 3 fetal demises occurred. All fetal demises were temporally and etiologically unrelated with the endoscopies. Following colonoscopy, there was one voluntary abortion and one fetal demise, both also temporally and etiologically unrelated with the colonoscopy.

In 2010, a study focusing exclusively on colonoscopies during pregnancy was published [3]. This retrospective, controlled cohort study reported on the safety and efficacy of colonoscopy in 20 pregnant patients. These pregnant patients were matched 1:1 with 20 pregnant controls with the same indication for colonoscopy but who did not undergo colonoscopy due to pregnancy. The study group was also compared to the pregnancy outcomes of the American national average. The majority of colonoscopies were performed in the second trimester of pregnancy (n = 16), with only 2 colonoscopies performed in respectively the first and third trimester. The study group trended towards worse pregnancy outcomes like stillbirth, premature delivery, low birth weight, low APGAR score, congenital defects and infant death after live birth, compared to the American national average. These non-significant differences can be attributed to the underlying illness in the study group according to the authors. When compared to the control group as described above, the study group tended to have slightly better fetal outcomes compared to the control group in terms of premature delivery, low birth weight, APGAR scores, congenital defects, neonatal ICU stay, infant postpartum hospitalization and infant death after live birth.

### Description of the case reports

The 79 case reports describing 92 patients are summarized and categorized per trimester in Tables 1, 2 and 3.

**Table 1:** **First trimester fetal and maternal adverse events (wk 1–12)**

Indication	N	Maternal adverse events	Pregnancy outcome	Spontaneous abortion	Other fetal adverse events	Temporal relation with endoscopy?	Etiological relation with endoscopy?
***Sigmoidoscopy***							
*IBD & colitis other* [11-16]	6	None	Live birth (n = 6)	No (n = 6)	3 premature births (34, 28 and 25.5 wks)	No	No
*Malignancy* [17,18]	3 (in 2 pts)	None	Elective abortion (n = 2)	No (n = 2)	Elective abortion (unwanted pregnancies)	Yes (n = 2)	No
*Volvulus and incarcerated uterus* [19]	1	None	No pregnancy losses	No (n = 1)	Not reported	No	No
*Non-malignant colonic obstruction*	0	-	-	-	-	-	-
*Gastrointestinal bleeding* [20]	1	None	Incomplete abortion (n = 1)	Yes, incomplete abortion at 10.4 wks (n = 1)	-	Yes	Possible, abdominal pregnancy, laparotomy after sigmoidoscopy
***Colonoscopy***							
*IBD & colitis other* [21-27]	12	None	Live births (n = 11), stillbirth (n = 1)	No (n = 12)	2 premature births (32 and 33 wks), 1 stillbirth (22 wks)	Unclear, paper fails to show which outcome belongs to which patient	No, authors do not link adverse events to endoscopy
*Malignancy* [28-32]	5	Maternal death (n = 1), none (n = 4)	Live birth (n = 5)	No (n = 5)	3 premature births (33, 33.6 and 34 wks)	No	No
*Volvulus and incarcerated uterus*	0	-	-	-	-	-	-
*Non-malignant colonic obstruction* [33]	1	None	Live birth (n = 1)	No (n = 1)	None	No	No
*Gastrointestinal bleeding* [34,35]	3 (in 2 pts)	None	Live birth (n = 2)	No (n = 2)	None	No	No
**Total**	**32**						

IBD = Inflammatory Bowel Disease.

**Table 2:** **Second trimester fetal and maternal adverse events (wk 13–26)**

Indication	N	Maternal adverse events	Pregnancy outcome	Premature births	Other fetal adverse events	Temporal relation with endoscopy?	Etiological relation with endoscopy?
***Sigmoidoscopy***							
*IBD & colitis other* [36-41]	8 (in 6 pts)	None (n = 6)	Live birth (n = 5), not reported (n = 1)	Yes (n = 1), no (n = 4), not reported (n = 1)	Low birth weight (n = 2), not reported (n = 1)	No	No
*Malignancy* [42-47]	6	Maternal death (n = 2), unreported (n = 1), none (n = 3)	Live birth (n = 3), elective abortion (n = 2), fetal death (n = 1)	Yes (n = 3) all prostaglandin induced or elective caesarean section	Low birth weight (n = 3)	Yes (n = 3), no (n = 3)	Unlikely (n = 3)
*Volvulus and incarcerated uterus* [19,48,49]	7 (in 5 pts)	None	Live birth (n = 5)	None	Low birth weight (n = 1)	No	No
*Non-malignant colonic obstruction* [50]	1	None	Live birth (n = 1)	Yes (n = 1)	Vaginal delivery at 35 wks	No	No
*Gastrointestinal bleeding* [51]	1	None	Stillbirth (n = 1)	Yes (n = 1)	Fetal demise at 20 wks within several hours of surgery	Yes	Possible, however the patient also underwent emergency surgery and suffered from a massive hemorrhage
***Colonoscopy***							
*IBD & colitis other* [24,52-54]	6	None	Live birth (n = 5), stillbirth (n = 1)	Yes(n = 2), No (n = 4)	Unreported (n = 2), none (n = 4)	Unclear, paper fails to show which outcome belongs to which patient	Unclear, authors do not link adverse event (stillbirth) to endoscopy
*Malignancy* [55-60]	6	None (n = 3), maternal death postpartum (n = 2), disease progression postpartum (n = 1)	Live birth (n = 4), unreported (n = 1), fetal death at 26 wks(n = 1)	Yes (n = 3) at 30, 34 and 36 wks	Low birth weight (n = 3), neonatal care unit admittance postpartum (n = 2)	Yes, fetal death was within 1 week of colonoscopy, premature births no temporal relation with endoscopy	Probable, but fetal death most likely due to maternal deterioration because of cancer progression and sepsis
*Volvulus and incarcerated uterus* [61]	1	Not reported	Live birth (n = 1)	No	None	No	No
*Non-malignant colonic obstruction* [62]	1	Mother remained hospitalized for 50 days after delivery	Stillbirth (n = 1)	Yes (n = 1)	Evidence of spontaneous labour, physicians terminated the pregnancy at 15 wks	Yes	Probable, however colonic perforation was feared due to worsening distention of the bowel, not per se due to the LGE
*Gastrointestinal bleeding* [34,63]	2	None (n = 2)	Live birth (n = 1), not reported (n = 1)	No (n = 1), not reported (n = 1)	Not reported (n = 1), None (n = 1)	No	No
**Total**	**39**

**Table 3:** **Third trimester fetal and maternal complications (27–42 wks)**

Indication	N	Maternal adverse events	Pregnancy outcome	Premature birth	Fetal adverse events	Temporal relation with endoscopy?	Etiological relation with endoscopy?
***Sigmoidoscopy***							
*IBD & colitis other* [11,64,65]	3	None (n = 2), subtotal colectomy with ileostomy after delivery (n = 1)	Live birth (n = 3)	No (n = 1), Yes (n = 2)	Premature births (28 and 34 wks), low birth weight (1850 and 1054 g)	No (n = 2), yes (n = 1)	Likely, after sigmoidoscopy colonic perforation was suspected, this led to an emergency caesarean section.
*Malignancy* [66-70]	5	Not reported (n = 3), death 12 months after hemicolectomy (n = 1), 1,5 years after delivery discovery of pulmonary metastases (n = 1)	Live birth (n = 5)	Yes (n = 4), No (n = 1)	Premature births at 34, 34, 31 and 33 wks, all deliveries were elective, low birth weight reported (n = 2)	No	Unlikely
*Volvulus and incarcerated uterus* [49,71-73]	5 (in 4 pts)	None	Live birth (n = 4)	None	None	Yes (n = 1)	Unlikely
*Non-malignant colonic obstruction* [74,75]	2	None	Live birth (n = 1), not reported (n = 1)	Yes (n = 1)	Elective caesarean section (n = 1), Not reported (n = 1)	Yes (n = 1)	Unlikely
*Gastrointestinal bleeding*	0	-	-	-	-	-	-
***Colonoscopy***							
*IBD & colitis other* [52,76]	2	Intensive care unit admittance postpartum (n = 1), none (n = 1)	Live birth (n = 1), not reported (n = 1)	Yes (n = 1), not reported (n = 1)	Premature birth (32 wks) with low birth weight 2175 grams	No	Unlikely
*Malignancy* [77-84]	8	None (n = 4), maternal death after delivery due to disease progression (n = 4)	Live birth (n = 8)	Yes (n = 8)	Premature births by elective caesarean section (n = 4), spontaneous premature birth (n = 4)	Yes (n = 1), no (n = 7)	Unlikely
*Volvulus and incarcerated uterus*	0	-	-	-	-	-	-
*Non-malignant colonic obstruction* [85,86]	3 (in 2 pts)	None	Live birth (n = 1), live twin birth (n = 1)	Yes (n = 1)	Spontaneous premature birth of twins at wk 34	Yes	Possible, however nifedipine was also stopped around time of LGE
*Gastrointestinal bleeding* [87]	1	None	Elective termination at 34 wks	Yes	Not reported	No	Unlikely
**Total**	**29**						

### Indications for LGE

Roughly, five major indications for endoscopy could be distinguished: (1) IBD and other colitis, (2) malignancy, (3) volvulus or incarcerated uterus, (4) non-malignant colonic obstruction and (5) gastrointestinal bleeding.

### Adverse events related to LGE

All temporally and etiologically related adverse events identified from the case reports are summarized in Table 4.

**Table 4:** **Summary of adverse events (AEs) etiologically related to LGE**

	Week of LGE	Week of AE	Type of AE	Other intervention between LGE and AE	Likeliness relation
***Sigmoidoscopy***					
	10	10.4	Incomplete spontaneous abortion	Laparotomy	Possible
	20	20	Fetal death	Laparotomy	Possible
	28	28	Suspected perforation leading to emergency caesarean section	Laparotomy and caesarean section at same time	Likely
***Colonoscopy***					
	25	26	Fetal death	None	Probable
	15.2	15.3	Pregnancy termination by physicians	None	Probable
	34.0	34.1	Premature spontaneous labour	Nifedipine cessation	Possible

### First trimester

In the first trimester, 32 LGEs were performed in 30 patients. All complications following LGE in the first trimester are listed in Table 1. Three adverse events occurred within 1 week of the LGE. In one case report [20], the patient underwent sigmoidoscopy at gestational week 10 and the patient had an incomplete spontaneous abortion at 10.4 weeks. The patient suffered from severe rectal bleeding due to a heterotopic, abdominal pregnancy protruding the terminal ileum. This adverse event could possibly be attributed to the LGE, because this patient also underwent laparotomy after the LGE and suffered from severe gastrointestinal bleeding. The other two temporally related adverse events in the first trimester were both elective abortions, and were therefore classified as etiologically unrelated to the LGE [17,18].

### Second trimester

In the second trimester, 39 endoscopies were performed in 35 patients. All complications following LGE in the second trimester are listed in Table 2. Six adverse events occurred within one week of LGE. Three cases reported three fetal deaths within one week of endoscopy. In the first case [51], the patient suffered from massive hematochezia due to multiple bleeding foci in the cecum and terminal ileum and underwent laparotomy shortly after colonoscopy. Fetal demise was evident several hours after surgery. This adverse event is possibly related to the LGE. The second patient was diagnosed with an advanced stage of colorectal carcinoma with liver metastases and ascites during pregnancy. After colonoscopy, the patient deteriorated rapidly and seven days after endoscopy fetal death was observed by ultrasonography. The mother died within 2 weeks after delivery [55]. This adverse event can probably be related to the LGE. The third case demonstrated a patient with progressive colonic distension caused by colonic pseudo-obstruction (Ogilvie’s syndrome). After colonoscopy, radiologic studies showed no evidence of colonic perforation, but the day after colonoscopy the abdominal distension progressed further, the patient went into spontaneous labor and the physicians decided to terminate the pregnancy [62]. This adverse event could also probably be related to the LGE. Two patients diagnosed with colorectal adenocarcinoma during pregnancy underwent elective abortion within one week of LGE in gestational week 16 and 20 [42,43] and in one patient labor was induced with prostaglandin in gestational week 26 [44]. These three adverse events were therefore classified as unlikely related to the LGE.

### Third trimester

In the third trimester, 27 patients underwent 29 endoscopies. All complications following LGE in the third trimester are listed in Table 3. Four case reports demonstrated adverse events within one week of endoscopy. These four cases were likely related in one, possibly related in one and unlikely related in two of the cases. The first case describes a patient who was diagnosed with ulcerative colitis upon sigmoidoscopy in the sixth week of pregnancy. In the 28th week of pregnancy she exhibited signs of exacerbation and she underwent another sigmoidoscopy with biopsies. Following the second sigmoidoscopy, colonic perforation was suspected and an emergency caesarean section and exploratory laparotomy was performed. No colonic perforation was seen intraoperatively [11]. A live, healthy baby of 1054 g was delivered. This adverse event was classified as likely to be related to the LGE. The second patient was 33 weeks pregnant with twins, when she underwent two subsequent colonoscopies for the treatment and decompression of acute colonic pseudo-obstruction. She was already being treated with nifedipine upon presentation for inhibition of premature contractions, and nifedipine was stopped upon hospital admission. One day after the last colonoscopy at gestational week 34, she went into spontaneous labor and delivered healthy twins [85]. This adverse event is possibly related to the LGE. The third patient underwent sigmoidoscopy because of abdominal pain and distention in the 34th gestational week. Upon endoscopy, the splenic flexure appeared necrotic and the patient immediately underwent laparotomy with an emergency caesarean section [74]. This adverse event is unlikely related to the LGE. The fourth patient was diagnosed with a malignancy of unknown origin, and in the metastatic workup a colonoscopy was performed in gestational week 32. A poorly differentiated signet cell adenocarcinoma of the transverse colon was found, and after 4 days of dexamethasone administration for fetal lung maturation an elective caesarean section was performed [77]. This adverse event was unlikely related to the LGE.

One case report and one case series did not report at what gestational week the LGE was performed and were therefore not categorized. These case reports describe three pregnant women with IBD who underwent sigmoidoscopy for IBD disease assessment. One woman delivered a live baby of 1008 gram prematurely at 28.1 weeks [88]. A temporal relation was not found, and the authors do not link this adverse event to the sigmoidoscopy. In the case series, 2 out of 5 women underwent sigmoidoscopy, and one woman delivered a live baby prematurely. It is not reported if this woman underwent LGE [89].

### Sensitivity analysis

A sensitivity analysis was performed by elongating the time span for the temporal relation between adverse events and the LGE. Initially, all adverse events were temporally related to the LGE if they occurred within one week after the LGE, however this analysis will classify all adverse events within three weeks of the LGE as temporally related. In the first trimester, this approach yielded no extra temporally related adverse events. In the second trimester, one additional temporally related adverse event was detected. In this case, the mother was diagnosed with advanced colorectal carcinoma during pregnancy and died together with the fetus two weeks after hospital admission around gestational week 23 [45]. This adverse event was unlikely to be related to the LGE. Finally, in the third trimester another seven temporally related adverse events were detected. Six premature deliveries were unlikely related to the LGE, as they were all elective caesarean sections [76,78-80,87] or induced labor [66]. The seventh patient suffered from ulcerative colitis and underwent LGE for assessment of disease activity in gestational week 32. Endoscopy showed the colon to be severely inflamed and two weeks later the patient delivered a premature baby of 1850 grams [64]. This adverse event is classified as probably related to the LGE.

## Discussion

The objective of this systematic review was to assess the risk of LGE in all trimesters of pregnancy.

Three retrospective cohort studies investigated the safety of LGE during pregnancy. Of these, two studies describe the same study population, and report no difference in birth outcomes and adverse events between the study and the control group. None of the reported fetal and maternal adverse events showed a temporal or an etiological relation with the LGE [8,10]. Although these studies report no adverse events related to LGE, it remains unclear in which trimester the LGE was performed.

The third study [3], on which the recent endoscopy guidelines [9] seem to be based, focuses exclusively on colonoscopies during pregnancy. The authors conclude that colonoscopies during pregnancy are probably safe to perform, but limit their conclusion to the second trimester because of insufficient data in the first and third trimester. Prior to this study in 2010, the authors identified 17 case reports on colonoscopy during pregnancy and add these data to their own conclusion that there is still insufficient evidence to claim safety of colonoscopy in each trimester [3].

Overall, this systematic review identified 79 case reports, describing 100 LGE’s in 92 patients. In total six (6.0%) temporally and etiologically related adverse events were found.

Out of these 79 case reports 42 case reports described 51 colonoscopies in 49 patients during pregnancy, distributed equally across the trimesters (21, 16 and 14 colonoscopies in trimester 1, 2 and 3, respectively). Three temporally and etiologically related adverse events occurred in these 49 patients (6.1%), of which 1 occurred in the third trimester [85] and was possibly related and 2 occurred in the second trimester [55,62] and were probably related to the colonoscopy (see Table 2 and 3). Although the evidence level of these case reports is low, these data suggest colonoscopy during pregnancy is probably safe to perform. This finding is in agreement with the primary conclusion of the included studies. However, the data from our included case reports in fact suggests colonoscopy to be of similar low risk in each trimester. In addition, we identified 37 case reports, describing 49 sigmoidoscopies in 43 patients. In this subset of patients, also three temporally and etiologically related adverse events occurred in these 43 patients (7.0%), of which one occurred in the first [20] and one in the second trimester [51] and were both possibly related, and one in the third trimester [11] and was likely related to the sigmoidoscopy.

Furthermore, in our view, postponing LGE during pregnancy or even until after pregnancy might hamper the patient and the pregnancy more than the LGE itself. A diagnostic delay will inevitably induce an unwanted therapeutic delay, and therefore the risks of LGE during pregnancy must be weighed against the expected benefits. Consequently, elective endoscopies (e.g. for screening purposes) should be deferred until after pregnancy.

Safety research during pregnancy is always a challenging field, as prospective studies are rarely, and experimental studies are almost never performed. Therefore, we rely on retrospective studies and case series to support our conclusions and guidelines. Although the evidence in this systematic review is anecdotal and more controlled studies are needed, this review appears to be the most extensive overview of available studies on this subject.

The major limitation of this exhaustive systematic review is the lacking of a solid control group for the summarized case reports. Furthermore, the majority of case reports describe severely ill patients in whom the true effect of LGE during pregnancy is hard to untangle. In addition, none of the case reports primarily aimed to describe the effect of LGE during pregnancy, rendering these effects subject to our interpretation. Type of bowel preparation and sedation are not mentioned in the majority of included case reports, and their effects cannot be taken into consideration. Also, mild and more subtle adverse events due to LGE could have been easily missed. We therefore focused on serious adverse events like spontaneous abortion, stillbirth and premature delivery.

## Conclusion

In conclusion, we underline that LGE should only be performed during pregnancy when strongly indicated and is probably of low risk. Postponing LGE during pregnancy to the second trimester or puerperium however, is unnecessary and in most cases unwanted because of the therapeutic delay which might hamper the pregnancy outcomes more than the LGE itself.

## Additional file

Additional file 1:
**Search strategy.**
