# Supplementary material for: Does lower gastrointestinal endoscopy during pregnancy pose a risk for mother and child? – a systematic review
Source: BMC Gastroenterol. 2015 Feb 12;15:15. doi: 10.1186/s12876-015-0244-z (PMC4339426; doi:10.1186/s12876-015-0244-z)
Supplement: Additional file 1: — Search strategy. [file 12876_2015_244_MOESM1_ESM.docx]

**Additional file 1**

**Embase.com**

(pregnancy/exp OR 'pregnant woman'/de OR 'pregnancy complication'/exp OR 'pregnancy outcome'/de OR 'fetus death'/de OR stillbirth/de OR 'immature and premature labor'/exp OR (childbear* OR gestation OR pregnan* OR abort* OR ((fetus OR fetal OR foetus OR foetal OR intrauterine) NEAR/3 (death OR loss OR dead)) OR stillbirth* OR stillborn OR (still NEXT/1 (birth* OR born*)) OR ((immature OR premature) NEAR/3 labor)):ab,ti) AND ('digestive tract endoscopy'/de OR 'gastrointestinal endoscopy'/de OR 'intestine endoscopy'/de OR colonoscopy/de OR rectoscopy/de OR sigmoidoscopy/de OR 'colon cancer'/exp OR 'occult blood'/de OR colitis/de OR (((digestive OR gastrointestin* OR intestin* OR colon* OR rectum OR rectal OR sigmoid*) NEAR/3 (endoscop* OR scopy)) OR proctosigmoidoscop* OR rectosigmoidoscop* OR ((colon OR colorect* OR rectal OR rectum OR sigmoid) NEAR/3 (cancer* OR carcinom* OR tumo* OR neoplas*)) OR colitis OR (gastrointestin* NEAR/3 hemorrhage*) OR (bowel NEAR/3 inflam*) OR IBD OR melena OR melaena ):ab,ti) NOT ([Conference Abstract]/lim OR [Conference Paper]/lim OR [Letter]/lim OR [Note]/lim OR [Editorial]/lim OR [Erratum]/lim) NOT ([animals]/lim NOT [humans]/lim)

**Medline (OvidSP)**

(exp pregnancy/ OR "pregnant women"/ OR exp "pregnancy complications"/ OR "fetus death"/ OR exp "Infant, Premature"/ OR (childbear* OR gestation OR pregnan* OR abort* OR ((fetus OR fetal OR foetus OR foetal OR intrauterine) ADJ3 (death OR loss OR dead)) OR stillbirth* OR stillborn OR (still ADJ (birth* OR born*)) OR ((immature OR premature) ADJ3 labor)).ab,ti.) AND (exp "Endoscopy, Digestive System"/ OR exp "Colonic Neoplasms"/ OR "occult blood"/ OR exp colitis/ OR (((digestive OR gastrointestin* OR intestin* OR colon* OR rectum OR rectal OR sigmoid*) ADJ3 (endoscop* OR scopy)) OR proctosigmoidoscop* OR rectosigmoidoscop* OR ((colon OR colorect* OR rectal OR rectum OR sigmoid) ADJ3 (cancer* OR carcinom* OR tumo* OR neoplas*)) OR colitis OR (gastrointestin* ADJ3 hemorrhage*) OR (bowel ADJ3 inflam*) OR IBD OR melena OR melaena ).ab,ti.) NOT (Congresses OR Letter OR Editorial OR published Erratum).pt. NOT (exp animals/ NOT humans/)

**Cochrane**

((childbear* OR gestation OR pregnan* OR abort* OR ((fetus OR fetal OR foetus OR foetal OR intrauterine) NEAR/3 (death OR loss OR dead)) OR stillbirth* OR stillborn OR (still NEXT/1 (birth* OR born*)) OR ((immature OR premature) NEAR/3 labor)):ab,ti) AND ((((digestive OR gastrointestin* OR intestin* OR colon* OR rectum OR rectal OR sigmoid*) NEAR/3 (endoscop* OR scopy)) OR proctosigmoidoscop* OR rectosigmoidoscop* OR ((colon OR colorect* OR rectal OR rectum OR sigmoid) NEAR/3 (cancer* OR carcinom* OR tumo* OR neoplas*)) OR colitis OR (gastrointestin* NEAR/3 hemorrhage*) OR (bowel NEAR/3 inflam*) OR IBD OR melena OR melaena ):ab,ti)

**Web-of-science**

TS=(((childbear* OR gestation OR pregnan* OR abort* OR ((fetus OR fetal OR foetus OR foetal OR intrauterine) NEAR/3 (death OR loss OR dead)) OR stillbirth* OR stillborn OR (still NEAR/1 (birth* OR born*)) OR ((immature OR premature) NEAR/3 labor))) AND ((((digestive OR gastrointestin* OR intestin* OR colon* OR rectum OR rectal OR sigmoid*) NEAR/3 (endoscop* OR scopy)) OR proctosigmoidoscop* OR rectosigmoidoscop* OR ((colon OR colorect* OR rectal OR rectum OR sigmoid) NEAR/3 (cancer* OR carcinom* OR tumo* OR neoplas*)) OR colitis OR (gastrointestin* NEAR/3 hemorrhage*) OR (bowel NEAR/3 inflam*) OR IBD OR melena OR melaena )) NOT ((animal* OR mouse* OR mice OR rat OR rats OR horse* OR monkey* OR lamb* OR sheep OR calf OR cattle OR dog OR dogs OR rodent* OR rabbit*) NOT (human* OR patient* OR mother* OR baby OR babies OR infant* OR child*)))

**PubMed publisher**

((childbear*[tiab] OR gestation[tiab] OR pregnan*[tiab] OR abort*[tiab] OR ((fetus[tiab] OR fetal[tiab] OR foetus[tiab] OR foetal[tiab] OR intrauterine[tiab]) AND (death[tiab] OR loss[tiab] OR dead[tiab])) OR stillbirth*[tiab] OR stillborn[tiab] OR still birth*[tiab] OR still born*[tiab] OR immature labor*[tiab] OR premature labor[tiab])) AND ((((digestive[tiab] OR gastrointestin*[tiab] OR intestin*[tiab] OR colon*[tiab] OR rectum[tiab] OR rectal[tiab] OR sigmoid*[tiab]) AND (endoscop*[tiab] OR scopy[tiab])) OR proctosigmoidoscop*[tiab] OR rectosigmoidoscop*[tiab] OR ((colon[tiab] OR colorect*[tiab] OR rectal[tiab] OR rectum[tiab] OR sigmoid[tiab]) AND (cancer*[tiab] OR carcinom*[tiab] OR tumo*[tiab] OR neoplas*[tiab])) OR colitis[tiab] OR (gastrointestin*[tiab] AND hemorrhage*[tiab]) OR (bowel[tiab] AND inflam*[tiab]) OR IBD[tiab] OR melena[tiab] OR melaena[tiab])) AND publisher[sb]

**Google Scholar**

gestation|pregnancy|pregnant |abortion|"fetal|foetal|intrauterine death|loss|dead" |stillbirth|stillborn "digestive|gastrointestinal|intestinal|colonic|rectum|rectal|sigmoid endoscopy|scopy"|proctosigmoidoscopy|rectosigmoidoscopy|colonoscopy
